# Genome Sequences of 64 Non-O157:H7 Shiga Toxin-Producing *Escherichia coli* Strains

**DOI:** 10.1128/genomeA.01067-15

**Published:** 2015-10-01

**Authors:** Magaly Toro, Guojie Cao, Lydia Rump, T. G. Nagaraja, Jianghong Meng, Narjol Gonzalez-Escalona

**Affiliations:** a Division of Microbiology, Center for Food Safety and Applied Nutrition, Food and Drug Administration, College Park, Maryland, USA; b Joint Institute for Food Safety and Applied Nutrition (JIFSAN), University of Maryland, College Park, Maryland, USA; c Department of Nutrition and Food Science, University of Maryland, College Park, Maryland, USA; d Department of Diagnostic Medicine and Pathobiology, College of Veterinary Medicine, Kansas State University, Manhattan, Kansas, USA

## Abstract

Shiga toxin-producing *Escherichia coli* (STEC) strains are human pathogens. Although >400 non-O157 serotypes have been involved in human disease, whole-genome sequencing information is missing for many serotypes. We sequenced 64 STEC strains comprising 38 serotypes, isolated from clinical sources, animals, and environmental samples, to improve the phylogenetic understanding of these important foodborne pathogens.

## GENOME ANNOUNCEMENT

In 1982, the first Shiga toxin-producing *Escherichia coli* (STEC) serotype, *E. coli* O157:H7, was associated with mild to severe human disease and outbreaks (1). Since then, its diversity has been widely studied (2). However, not only O157:H7 has been implicated with human diseases, as >400 STEC serotypes have also been found to be responsible for them (3). In the United States, six serogroups, O26, O45, O103, O111, O121, and O145, cause 70% of non-O157 STEC diseases (4). Although some of these serotypes have been described genetically (5), many more strains still have not been assigned to serogroups or sequenced.

Shiga toxin (Stx) is a cytotoxin similar to *Shigella dysenteriae* toxin type 1 (6); the two main *stx* gene variants are Shiga toxin 1 (*stx*_1_) and Shiga toxin 2 (*stx*_2_), which damage intestinal epithelial cells and kidneys, causing hemorrhagic colitis (HC) and hemolytic-uremic syndrome (HUS), respectively (7, 8). Other virulence factors carried by STECs include intimin (*eae*) and plasmid-borne enterohemolysin (*ehxA*), both of which contribute to severe disease in humans (6, 9).

Sixty-four STEC cultures were grown aerobically overnight in tryptic soy agar (TSA) at 37°C, and then DNA was extracted using the DNeasy blood and tissue kit (Qiagen, Valencia, CA). Libraries were prepared using the Nextera XT kit (Illumina, San Diego, CA) and 1 ng of genomic DNA. Sequences were obtained with the MiSeq Illumina V2 (2 × 250 bp) or V3 kit (2 × 300 bp), according to the manufacturer’s instructions, and *de novo*-assembled sequences were generated using CLC Genomics Workbench version 7.6.1 (CLC bio, Germantown, MD, USA). Strains were sequenced to a coverage depth ranging from 34 to 118×. We used Ridom SeqSphere+ for *in silico* MLST analysis, and resulting sequences were annotated using the NCBI Prokaryotic Genomes Annotation Pipeline (PGAP) (http://www.ncbi.nlm.nih.gov/genome/annotation_prok).

These genomes varied from 4.7 to 5.4 Mb; the number of contigs per assembly ranged from 52 to 303 (data not shown). Most strains represented 27 previously described sequence types (STs); seven strains were novel STs (Table 1). Using the CGE server at the Technical University of Denmark (10), we ran *in silico* analyses of serotypes and for the presence of virulence genes (*stx*, *eae*, and *exhA*). Forty-eight strains (75%) carried *stx*_1_, and 41 strains (64%) carried *stx*_2._ A total of 25 strains (39%) carried both *stx* genes (Table 1). Twenty-five strains (39%) carried four different subtypes of *eae*: beta1, epsilon, gamma1, and theta2. The plasmid-borne *exhA* gene was detected in 52 strains (81%). The information we report here will help better understand the evolution of these emergent foodborne pathogens (non-O157 STECs) and improve the accuracy of food-related trace-back investigations outbreaks caused by these pathogens.

**TABLE 1:** Metadata for Shiga toxin-producing *Escherichia coli*

CFSAN no.	Isolate name	WGS accession no.	Serogroup/serotype		ST	*stx*_1_^a^	*stx*_2_^a^	*eae* subtype	*ehxA^a^*
			Reported	*In silico^a^*					
CFSAN026773	5750	LDDW00000000	O6	O6:H34	New1^b^	+^c^	+	−	+
CFSAN026776	2794-3	LGZO00000000	O15	O15:H27	5012	+	+	−	−
CFSAN026777	1245	LGZZ00000000	O22	O22:H8	446	+	+	−	+
CFSAN026778	748-1	LDDV00000000	O38	O38:H21	56	+	+	−	−
CFSAN026779	13950-1	LDDU00000000	O38	O38:H22	New2	−	+	−	+
CFSAN026781	4558-1	LGZW00000000	O74	O74:H42	5013	+	+	−	+
CFSAN026783	1235-1	LGZC00000000	O88	O88:H25	154	+	+	−	+
CFSAN026785	4162	LDDT00000000	O91	O91:H21	442	−	+	−	+
CFSAN026786	3712-2	LGZJ00000000	O96	O96:H19	New3	+	+	−	+
CFSAN026787	497	LDDS00000000	O96	O96:H19	1611	−	+	−	+
CFSAN026788	4	LDDR00000000	O111:NM	O111:H8	16	+	+	θ2	+
CFSAN026789	7726-1	LDDQ00000000	O111	O111:H8	16	+	+	θ2	+
CFSAN026790	7739-1	LDDP00000000	O111	O111:H8	16	+	+	θ2	+
CFSAN026791	7756-1	LDDO00000000	O111	O111:H8	16	+	+	θ2	+
CFSAN026792	8	LGZM00000000	O111	O111:H8	16	+	+	θ2	+
CFSAN026793	1598-2	LGZF00000000	O113	O113:H21	223	−	+	−	+
CFSAN026794	3517	LGZH00000000	O113	O113:H21	56	−	+	−	−
CFSAN026795	1238-1	LGZX00000000	O116	O116:H21	58	+	+	−	+
CFSAN026796	3536-3	LDDL00000000	O117	O117:H4	10	+	+	−	−
CFSAN026798	47	LGZK00000000	O121	O121:H19	655	−	+	ϵ	−
CFSAN026799	48	LDDK00000000	O121	O121:H19	655	−	+	ϵ	+
CFSAN026800	55	LGZT00000000	O121	O121:H19	655	−	+	ϵ	+
CFSAN026801	37	LDDJ00000000	O130:H11	O130:H11	297	−	+	−	+
CFSAN026802	492-1^d^	LDDI00000000	O130	O130:H38	New4	+	+	−	+
CFSAN026809	9388-1	LDDH00000000	O163	O163:H19	679	+	+	−	+
CFSAN026810	18917-1	LDDG00000000	O163	O163:H19	679	−	+	−	+
CFSAN026812	1044-1	LDDF00000000	O171	O171:H2	332	−	+	−	−
CFSAN026813	9042-1	LDDE00000000	O172	O172	660	−	+	ϵ	+
CFSAN026815	22	LDDD00000000	45:H2	O45:H2	17	+	−	ϵ	+
CFSAN026817	1.2622	LGZP00000000	O45:H12	O45:H16	2217	+	−	−	−
CFSAN026819	2	LGZQ00000000	103:H2	O103:H2	17	+	−	ϵ	+
CFSAN026821	3720-1	LGZR00000000	103	O103:H2	1967	+	−	ϵ	+
CFSAN026824	39	LGZL00000000	103	O103:H2	17	+	−	ϵ	+
CFSAN026825	40	LGZS00000000	103	O103:H2	1967	+	−	ϵ	+
CFSAN026826	54^d^	LDDC00000000	103	O103:H2	17	+	−	ϵ	+
CFSAN026828	2013-3-80A	LDDB00000000	103	O103:H2	1967	+	−	ϵ	+
CFSAN026820	20	LGZU00000000	103:H11	O103:H11	723	+	−	β1	+
CFSAN026830	2013-6-148B	LDDA00000000	104:H7	O104:H7	1817	+	−	−	+
CFSAN026831	2011-5-383-1	LHAA00000000	104	O104:H7	1817	+	−	−	+
CFSAN026835	12662-2	LDCZ00000000	109	O109	New5	+	−	−	+
CFSAN026836	15166-1	LDCY00000000	109	O109	New5	+	−	−	+
CFSAN026838	1939^d^	LDDN00000000	111	O111:H8	16	+	−	θ2	+
CFSAN026839	11189-1^d^	LDDM00000000	111	O111:H8	16	+	−	θ2	+
CFSAN026840	19^d^	LGZG00000000	111	O111:H8	16	+	−	θ2	+
CFSAN026842	1553-1^d^	LDCX00000000	121	O121:H7	New6	+	−	−	**−**
CFSAN026843	4709-1^d^	LGZN00000000	136	O136:H16	329	+	−	−	+
CFSAN026844	10314-1^d^	LDCW00000000	136	O136:H16	329	+	−	−	+
CFSAN026845	11182-2^d^	LDCV00000000	142	O142:H38	154	+	−	−	−
CFSAN026804	53	LGZD00000000	O145	O145	32	+	+	γ1	+
CFSAN026805	1234-1	LDCU00000000	O145	O145	32	+	+	γ1	+
CFSAN026846	2013-3-279 D^d^	LGZI00000000	145	O145	32	−	+	γ1	+
CFSAN026847	1^d^	LGZE00000000	145:NM	O145	32	+	−	γ1	+
CFSAN026848	2013-3-86 C^d^	LHAB00000000	145	O145	32	+	−	γ1	+
CFSAN026807	9916-1	LHCR00000000	− : H25	− : H25	58	+	+	−	+
CFSAN026808	1932	LHCS00000000	− : H25	− : H25	58	−	+	−	+
CFSAN026782	15484-1	LHCN00000000	O8:H19	O8:H19	2385	+	+	−	+
CFSAN026803	5344-1	LHCP00000000	O8:H49	O8:H49	111	+	+	−	+
CFSAN026775	2089-2	LHCX00000000	O8:H19	O8:H19	201	+	+	−	−
CFSAN026780	7712-3	LHCM00000000	O116:H49	O116:H49	2520	−	+	−	+
CFSAN026849	16118-2	LHCW00000000	O142:H38	O142:H38	154	+	−	−	−
CFSAN026806	5710-2	LHCQ00000000	O183:H18	O183:H18	657	+	+	−	+
CFSAN026797	6842-1	LHCO00000000	O185:H7	O185:H7	2387	−	+	−	−
CFSAN026811	1223-4	LHCT00000000	O134:H38	O134:H38	154	+	+	−	+
CFSAN026784	1027-4	LHCZ00000000	O22:H8	O22:H8	446	+	+	−	+

a These results were obtained by using the virulence finder and serotype options in the CGE server (http://cge.cbs.dtu.dk/services) (10).

b New indicates an ST not described previously/unknown.

c A plus sign indicates presence and a minus sign indicates absence.

d Strains sequenced using MiSeq Illumina V3 kit (2 × 300 bp). The remaining strains were sequenced using V2 (2 × 250 bp).

### Nucleotide sequence accession numbers. 

The draft genome sequences for these 64 STEC strains are available in GenBank and are listed in Table 1.
